# Factors Associated with Neutralizing Antibody Responses following 2-Dose and 3rd Booster Monovalent COVID-19 Vaccination in Japanese People Living with HIV

**DOI:** 10.3390/v16040555

**Published:** 2024-04-02

**Authors:** Isaac Ngare, Toong Seng Tan, Mako Toyoda, Takeo Kuwata, Soichiro Takahama, Eriko Nakashima, Naoya Yamasaki, Chihiro Motozono, Teruhisa Fujii, Rumi Minami, Godfrey Barabona, Takamasa Ueno

**Affiliations:** 1 Joint Research Center for Human Retrovirus Infection, Kumamoto University, 2-2-1 Honjo, Chuo-ku, Kumamoto 860-8555, Japan; ngareisaac@yahoo.com (I.N.); tstan@mgh.harvard.edu (T.S.T.); makotoyo@kumamoto-u.ac.jp (M.T.); tkuwata@kumamoto-u.ac.jp (T.K.); motozono@kumamoto-u.ac.jp (C.M.); barabona@kumamoto-u.ac.jp (G.B.); 2 Graduate School of Medical Sciences, Kumamoto University, 1-1-1 Honjo, Chuo-ku, Kumamoto 860-0811, Japan; 3 NHO, Kyushu Medical Center, 1-8-1 Jigyohama, Chuo-ku, Fukuoka 810-8563, Japan; takahama.soichiro.bt@mail.hosp.go.jp (S.T.); nakashima.eriko.rj@mail.hosp.go.jp (E.N.); minami.rumi.ad@mail.hosp.go.jp (R.M.); 4 Division of Transfusion Medicine, Hiroshima University Hospital, 1-2-3, Kasumi, Minami-ku, Hiroshima 734-8551, Japan; naoya64@hiroshima-u.ac.jp (N.Y.); teruchan@hiroshima-u.ac.jp (T.F.); 5 Joint Research Center for Human Retrovirus Infection, Kagoshima University, 8-35-1, Sakuragaoka, Kagoshima 890-8544, Japan

**Keywords:** SARS-CoV-2, neutralizing antibodies, COVID-19 vaccination, HIV, variants of concern

## Abstract

People living with HIV (PLWH) could be at risk of blunted immune responses to COVID-19 vaccination. We investigated factors associated with neutralizing antibody (NAb) responses against SARS-CoV-2 and variants of concern (VOCs), following two-dose and third booster monovalent COVID-19 mRNA vaccination in Japanese PLWH. NAb titers were assessed in polyclonal IgG fractions by lentiviral-based pseudovirus assays. Overall, NAb titers against Wuhan, following two-dose vaccination, were assessed in 82 PLWH on treatment, whereby 17/82 (20.73%) were classified as low-NAb participants. Within the low-NAb participants, the third booster vaccination enhanced NAb titers against Wuhan and VOCs, albeit to a significantly lower magnitude than the rest. In the multivariate analysis, NAb titers against Wuhan after two-dose vaccination correlated with age and days since vaccination, but not with CD4^+^ count, CD4^+^/CD8^+^ ratio, and plasma high-sensitivity C-Reactive protein (hsCRP). Interestingly, an extended analysis within age subgroups revealed NAb titers to correlate positively with the CD4^+^ count and negatively with plasma hsCRP in younger, but not older, participants. In conclusion, a third booster vaccination substantially enhances NAb titers, but the benefit may be suboptimal in subpopulations of PLWH exhibiting low titers at baseline. Considering clinical and immune parameters could provide a nuanced understanding of factors associated with vaccine responses in PLWH.

## 1. Introduction

The rollout of global COVID-19 vaccination raised concerns over the ability of people living with HIV (PLWH) to elicit optimal immune responses given their underrepresentation in preceding clinical trials [1,2]. A growing body of literature now suggests that although PLWH on antiretroviral therapy (ART) can mount antibody responses at a comparable magnitude to non-HIV-infected individuals [3,4], certain subpopulations of PLWH, such as those with suboptimal CD4^+^ count recovery, have shown attenuated antibody responses following two-dose vaccination [5,6,7]. Additionally, a substantial proportion of PLWH have shown no detectable antibody responses to a standard two-dose mRNA vaccination regimen [8]. As a result, PLWH have been categorized as a vulnerable population, with respect to severe COVID-19 [9], and have subsequently been recommended for updated monovalent and bivalent booster vaccination regimens [10]. However, whether subpopulations of PLWH exhibiting suboptimal antibody responses at baseline could optimally benefit from booster vaccination remains unclear. Thus, it is important to continue evaluating antibody responses in PLWH receiving booster vaccination regimens.

The evaluation of host factors associated with antibody responses to COVID-19 vaccination in PLWH on suppressive ART is increasingly challenging, in part, due to extensively heterogeneous immune reconstitution markers, chronic inflammation levels, and rates of co-morbidities in this population [11,12,13]. Investigations aiming to refine our understanding of host immune factors delineating certain subpopulations of PLWH presenting with suboptimal antibody responses to vaccination have been inconclusive thus far, and in some instances, conflicting. For instance, whereas HIV-related factors such as CD4^+^ count [8] and CD4^+^/CD8^+^ ratio [14] have been shown to associate with antibody responses in some cohorts, these factors did not impact antibody responses in others [4,15]. Additionally, clinical factors such as age and inflammation biomarkers [16], which have similarly shown a differential impact on antibody responses to vaccination, further highlight the complexity of predicting vaccine responses in PLWH. While the basis of these disparities remains unclear, they continue to impede efforts aimed at identifying most at-risk subpopulations of PLWH for targeted booster vaccination strategies. Hence, in this study, we investigated factors associating with neutralizing antibody (NAb) responses against SARS-CoV-2 and VOCs following two-dose and third booster monovalent COVID-19 vaccination in Japanese PLWH.

## 2. Materials and Methods

### 2.1. Study Participants

We enrolled PLWH on ART during scheduled routine HIV monitoring and ART refill visits in HIV care clinics at Kyushu Medical Center (*n* = 70) and Hiroshima University Hospital (*n* = 12) in Japan. Plasma samples after 2-dose COVID-19 mRNA vaccination were collected between September 2021 and January 2022, whereas samples after monovalent 3rd booster vaccination were collected between February and June 2022. HIV-related parameters of CD4^+^ count, CD4^+^/CD8^+^, HIV plasma viral load, and high-sensitivity C-Reactive protein (hsCRP), which are routinely monitored in the HIV care clinics from which the participants were recruited, were acquired at the point of sample collection. Pre-ART CD4^+^ count and pre-ART viral load data were also included.

### 2.2. Purification of Polyclonal IgG Fractions

Polyclonal IgG fractions were purified from participant plasma by spin columns equipped with protein A-conjugated silica beads (Cosmobio, cat # CSR-APK-10A, Tokyo, Japan), according to the manufacturer’s instruction. Briefly, 70 µL of heat-inactivated plasma was diluted with 210 µL of 1× phosphate-buffered saline (PBS) and passed through a 0.22 µM filter (Sigma-Aldrich, cat # SLGVR33RS, St. Louis, MI, USA) to minimize impurities in the end product. IgG fractions were eluted in 100 µL of manufacturer-provided elution buffer and quantified by a NanoDrop 2000 spectrophotometer (Thermo Fisher, Waltham, MA, USA) before storage at 4 °C for no longer than 3 months.

### 2.3. Production of Pseudotyped Viruses

SARS-CoV-2 and variant of concern (VOC) spike-bearing pseudoviruses were constructed by co-transfecting 293T cells (ATCCs), with modified versions of plasmids encoding spike and a lentiviral backbone, as previously reported [17]. In brief, spike-encoding plasmids (kindly provided by T. Kuwata [18]) were modified by truncating the last 19 amino acids from the C-terminal end of the cytoplasmic tail to create plasmids designated as SARS-CoV-2 or VOC-Spike CΔ19. The lentiviral backbone, pSG3_ΔENVΔNef_-Luc2-IN/HiBit (kindly provided by K. Tokunaga), was previously modified by inserting a HiBiT peptide tag sequence and a luciferase reporter gene [19]. The 2 modified plasmids were then used to co-transfect overnight-seeded 293T cells at a concentration of 1000 ng and 25 ng for pSG3_ΔENVΔNef_-Luc2-IN/HiBit and SARS-CoV-2/VOC-Spike CΔ19, respectively. Upon 48 h of incubation at 37 °C and 5% CO_2_, DNase I was added to the culture supernatant to degrade any unutilized plasmid. The culture supernatant was then passed through 0.45 µM filters (Sigma-Aldrich, cat # SLHVJ13SL, St. Louis, MI, USA) and aliquots thereof stored at −80 °C. Viral titers were quantified as a function of HiBiT-generated luminescence and normalized by the corresponding level of HIV p24 antigen (produced by the HIV-based proviral backbone), as described previously [20].

### 2.4. Target Cell Preparation

Target cells expressing SARS-CoV-2 surface receptors were prepared as previously described [17]. In brief, 293T cells cultured in Dulbecco’s Modified Eagle Medium (DMEM) (Thermo Fisher, cat # 041-29775, Waltham, MA, USA) enriched with 10% fetal bovine serum (FBS) (Sigma Aldrich, cat # 172012, St. Louis, MI, USA) were co-transfected with 500 ng of pCXN-ACE2 and 250 ng of pC-TMPRSS2 (kindly provided by K. Tokunaga [21]), and incubated at 37 °C and 5% CO_2_ for 48 h. The cells were then trypsinized 15 min prior to utilization in the ensuing polyclonal IgG neutralization assay.

### 2.5. Polyclonal IgG Neutralization Assay

NAb titers against SARS-CoV-2 and VOC spike in participant-derived polyclonal IgG fractions were assessed by previously developed HIV neutralization assays [22], subject to minor modifications. Briefly, participant-derived IgG was 3-fold serially diluted on a 96-well plate from starting concentrations of 100 µg/mL or 300 µg/mL. Pseudoviruses were thawed to 37 °C and added at a concentration of 3 ng/well of the proviral backbone p24 antigen level and incubated at 37 °C and 5% CO_2_. One hour after incubation, freshly trypsinized target cells (293T/ACE2/TMPRSS2) were added to the IgG/pseudovirus complex at a density of 22,000 cells/well and incubated for 48 h. A luciferase substrate (ONE-Glo, Promega, cat # E6130, Madison, MI, USA) equipped with a lysis agent was added to the culture supernatant in accordance with the manufacturer’s instructions. IgG neutralization at each dilution was calculated as a percentage reduction in luminescence in the IgG + pseudovirus wells relative to the pseudovirus-only wells. IgG NAb titers were expressed as IC_50_ values calculated on a dose–response curve fit with a non-linear function. An IgG sample of known NAb titer was used as a positive control to ensure consistency in conditions between assay runs.

### 2.6. Statistical Analysis

Bivariate and multivariate Spearman correlation analyses were performed by IBM SPSS Statistics, version 26 (Chicago, IL, USA). Nonparametric partial correlation analysis was performed to adjust for confounders of NAb titers. The Wilcoxon signed-rank test and Mann-Whitney U test were used to compare differences between paired populations and unpaired populations, respectively. All tests were two-tailed, and *p*-value of <0.05 was considered statistically significant.

## 3. Results

### 3.1. Cohort Characteristics

A total of 82 PLWH who reported no history of COVID-19 diagnosis were included in this analysis. Most participants, 48/67 (71.6%), received two doses of BNT162b2 (Pfizer, New York, NY, USA), while the rest 19/67 (28.4%) received two doses of mRNA-1273 (Moderna, Cambridge, MA, USA). The participants, who were majority male 80/82 (97.5%), had a median (IQR) age of 48 (40–56) years. All participants were on ART, although the plasma viral loads of 5 (6.3%) participants were above the detection limit (defined as >50 copies/mL). The median (IQR) CD4^+^ count at the time of sample collection was 470 (314–643) cells/µL. By the close of sample collection in June 2022, a total of 28 two-dose vaccinees (34%) had received a monovalent third booster vaccine dose. We, however, focused our analysis on a subset of 18 participants whose sampling time point after the third booster vaccination matched the sampling time point after two-dose vaccination (Table 1). Within the booster vaccinees, 12/15 (80%) received mRNA-1273, whereby the majority 10/15 (66.7%) received a booster dose by a different manufacturer from the first two doses. 

### 3.2. Neutralizing Antibody Titers against SARS-CoV-2 following 2-Dose Vaccination in PLWH

To assess the NAb titers of antisera, we employed a lentiviral reporter assay system pseudotyped by SARS-CoV-2 spike protein, as previously reported [19]. A version of spike protein which had been modified by truncating 19 amino acids from the C terminal end (CΔ19 spike) was used. Consistent with previous reports [23,24], the CΔ19 spike showed substantial enhancement in pseudovirus infectivity compared to its native form (Supplementary Figure S1). When we tested three representative plasma samples for unspecific pseudovirus inhibition, they all uniformly inhibited the infection of lentiviruses pseudotyped by SARS-CoV-2 (Wuhan) spike, including two pseudotyped by vesicular stomatitis virus G protein (VSV-G) and murine leukemia virus envelope (MuLV), which are of zoonotic extraction (Supplementary Figure S1). The confirmed unspecific inhibition activity targeting the proviral backbone likely emanated from residual ART in the participants’ plasma, which has been shown to indiscriminately inhibit lentiviral-based pseudoviruses [25,26]. Therefore, in order to abrogate unspecific inhibition by residual ART, we purified polyclonal IgG, which has been shown to be the dominant isotype in plasma anti-spike antibody responses [27]. Purified polyclonal IgG fractions did not show unspecific inhibition of pseudoviruses bearing the VSV-G and MuLV envelope but exhibited neutralizing activity specific for Wuhan spike, against which the participants had been recently vaccinated. Consequently, all subsequent assessments of pseudovirus neutralization activity were performed using purified polyclonal IgG antibody fractions and expressed as IC_50_ values.

**Figure 1:**
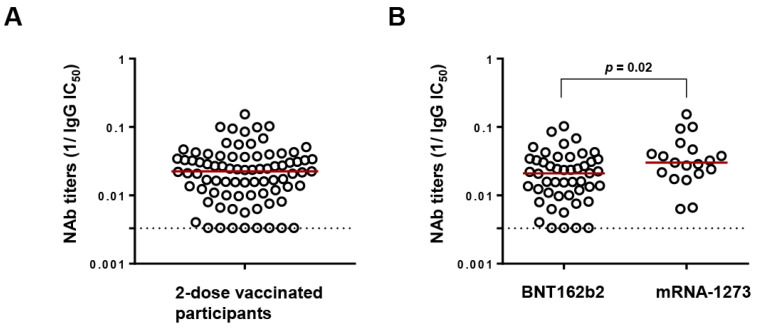
Neutralizing antibody (NAb) titers following 2-dose vaccination. (**A**) Distribution of NAb titers against Wuhan (expressed as reciprocal IC_50_) of all the study participants (*n* = 82). The red line denotes the median NAb titers while the dotted line represents the limit of detection (300 µg/mL (reciprocal = 0.003)). (**B**) Comparison of NAb titers between recipients of BNT162b2 (Pfizer) and mRNA-1273 (Moderna) by Mann–Whitney ranked U test.

Following two-dose vaccination, NAb titers against Wuhan were determined in the polyclonal IgG fractions of participants at a median (IQR) IC_50_ of 44.9 (27.4–94.9) µg/mL (Figure 1A). However, recipients of BNT162b2 exhibited significantly lower NAb titers than mRNA-1273 recipients (median IgG IC_50_ of 47.8 versus 33.2 µg/mL, *p* = 0.02), in agreement with reports from cohorts of PLWH and HIV uninfected people [28,29] (Figure 1B). A low NAb titer threshold was defined by identifying the upper IgG IC_50_ value of the lowest quartile (>100 µg/mL). As a result, 17/82 (20.73%) participants were considered to have low NAb titers, including 8 whose titers were below the detection limit. Among the 17 low-NAb participants, 13 had undetectable HIV viral loads at the time of sample collection, while 1 participant who had recently initiated ART had a viral load of 330,000 copies/mL. The median (IQR) age, CD4^+^ count, and CD4^+^/CD8^+^ ratio of the low-NAb participants were 58 (47–68) years, 301 (185–494) cells/µL, and 0.4 (0.2–1.1), respectively.

### 3.3. Neutralizing Antibody Titers against SARS-CoV-2 and Variants of Concern following Monovalent 3rd Booster Vaccination in PLWH

**Figure 2:**
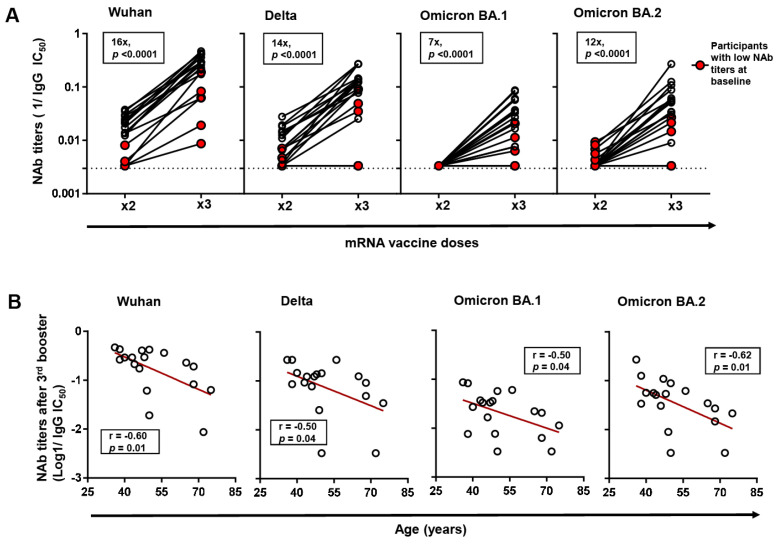
Changes in neutralizing antibody (NAb) titers against Wuhan and variants of concern (VOCs) following 3rd booster vaccination. (**A**) Changes in NAb titers against Wuhan and VOCs (Delta, Omicron BA.1, and Omicron BA.2) following 2-dose and 3rd booster vaccination in a subset of 3rd booster vaccinated participants (*n* = 18). Changes in the NAb titers of previously identified low-NAb-titer participants (*n* = 5) are highlighted in red. The *p*-values were computed by Wilcoxon ranked test. Median fold changes in NAb titers, alongside *p*-values, are indicated within each panel. (**B**) Spearman correlation between age and NAb titers against Wuhan, and VOCs following 3rd booster vaccination. The line of best fit (red line) was generated by linear regression.

Further, we analyzed the impact of third booster vaccination on NAb titers against Wuhan and other VOCs (Delta, Omicron BA.1 and Omicron BA.2) in a subset of 18 third-booster vaccinees in which 5/18 (27.8%) had been previously classified as low-NAb participants. Between the low-NAb participants and the rest, the days since two-dose and third booster vaccination were not significantly different. First, we established that third booster vaccination significantly enhanced NAb titers against Wuhan by a median fold of 16 (*p* = 0.0001). This boosting effect was observed in all participants, although low-NAb participants exhibited significantly lower NAb titers despite a comparable fold increase with the rest (Figure 2A). Similarly, the third booster vaccination enhanced NAb titers against all the VOCs tested by the following median folds: Delta (14-fold), Omicron BA.1 (7-fold), and Omicron BA.2 (12-fold) (*p* < 0.0001). Consistent with observations from Wuhan neutralization, low-NAb participants recorded significantly lower NAb titers against VOCs, and in two participants, titers showed no detectable change despite third booster vaccination.

### 3.4. Factors Associated with Neutralizing Antibody Titers following COVID-19 Vaccination in PLWH

Next, we investigated how HIV and vaccine-related variables correlated with polyclonal IgG NAb titers against Wuhan spike following two-dose vaccination. In the bivariate analysis, we found that the parameters of age (*r* = −0.42, *p* = 0.001), CD4^+^ count at sample collection (*r* = 0.28, *p* = 0.01), and days since two-dose vaccination (*r* = −0.45, *p* = 0.001) significantly correlated with NAb titers, whereas those of pre-ART viral load, pre-ART CD4^+^ count, and hsCRP at sample collection did not correlate (Table 2). Variables that correlated moderately or strongly with NAb titers, and at a statistical significance of *p* < 0.1 (age, CD4^+^ count, pre-ART viral load, and days since two-dose vaccination), were adjusted for via a nonparametric partial Spearman correlation analysis. The resulting adjusted Spearman correlations revealed that only age (*r* = −0.33, *p* = 0.007) and days since two-dose vaccination (*r* = −0.34, *p* = 0.005) independently correlated with NAb titers (Table 2). Given previous reports showing diminished humoral responses at lower CD4^+^ counts in PLWH [5], we also separately analyzed participants with a CD4^+^ count in the subnormal range (≤500 cells/µL), but the correlation with NAb titers lost statistical significance in the multivariate analysis as well.

Further, we analyzed factors correlating with NAb titers following the 3rd booster vaccination within the subset of 18 booster vaccinees. Consistent with observations after two-dose vaccination, we found that age, but not other host factors, negatively correlated with NAb titers against Wuhan (*r* = −0.59, *p* = 0.009) following third booster vaccination (Figure 2B). The correlation was also observed across all the VOCs investigated.

### 3.5. Factors Associated with Neutralizing Antibody Titers following COVID-19 Vaccination in Different Age Subgroups of PLWH

**Figure 3:**
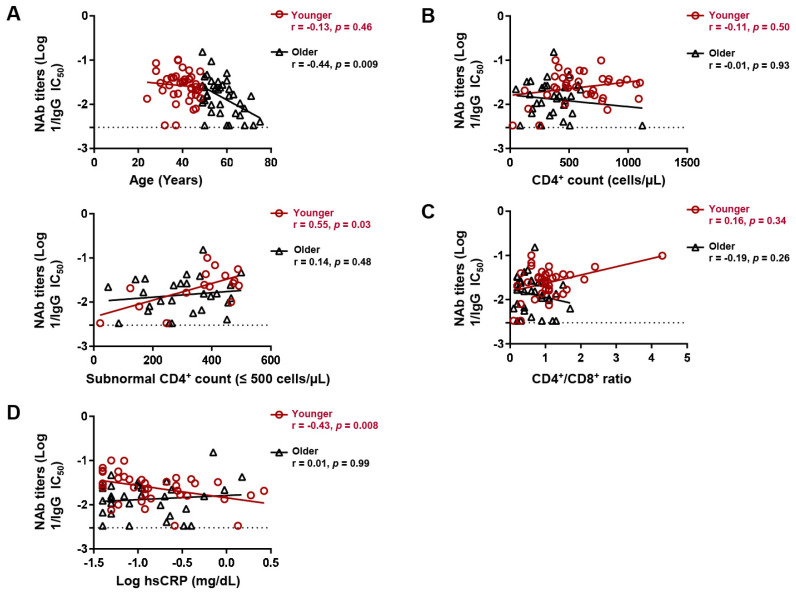
Age subgroup differences in factors associating with neutralizing antibody (NAb) titers following 2-dose vaccination. Spearman correlation between (**A**) age, (**B**) CD4^+^ count, (**C**) CD4^+^/CD8^+^ ratio, and (**D**) hsCRP levels and IgG NAb titers against Wuhan in younger (≤48 years) and older (>48 years) participants. hsCRP; high sensitivity C-reactive protein. Lines of best fit (black and red) were generated by linear regression.

Despite age being the only host correlate of NAb titers after two-dose vaccination, we noticed that this correlation was biphasic in that it was more prominent (*r* = −0.44, *p* = 0.009) in the upper 50th percentile of age and relatively diminished (*r* = −0.13, *p* = 0.46) in the lower 50th percentile (Figure 3A). We thus stratified the participants at the median age (48 years) and separately analyzed the correlates of NAb titers in the younger (≤48 years old, *n* = 42) and older (>48 years old, *n* = 40) subgroups. There was no significant difference in the proportion of BNT162b2 recipients between the younger and older age subgroups (69.7% versus 74.5%, *p* = 0.79). While the CD4^+^ count did not correlate with NAb titers in either subgroup, a CD4^+^ count within the subnormal range (≤500 cells/µL) positively correlated with NAb titers in the younger (*r* = 0.55, *p* = 0.029), but not older, subgroup (*r* = 0.14, *p* = 0.47) (Figure 3B). However, the CD4^+^/CD8^+^ ratio did not significantly correlate with NAb titers in either subgroup (Figure 3C). Consistent with subnormal CD4^+^ count, levels of hsCRP, which is a marker of inflammation [30], negatively correlated with NAb titers in the younger (*r* = −0.43, *p* = 0.008), but not older (*r* = −0.01, *p* = 0.998), subgroup (Figure 3D). hsCRP did not correlate with age in either subgroup.

## 4. Discussion

PLWH remain vulnerable to severe COVID-19, and identifying the most at-risk subpopulations remains a key objective. Our study analyzes factors associated with NAb responses in the polyclonal IgG fractions of Japanese PLWH receiving two-dose and third booster COVID-19 mRNA vaccination. We demonstrate that a subpopulation with low NAb titers against SARS-CoV-2, identified after two-dose vaccination, exhibits similarly low NAb titers against VOCs after the third booster vaccination. Additionally, different age subgroupings exhibited differential host factor associations with NAb titers following two-dose vaccination.

In this study, 17/82 (20.7%) participants were classified as low-NAb participants. However, we did not find any significant differences in markers of immune reconstitution (CD4^+^ count and CD4^+^/CD8^+^ ratio) between the low NAb titers subpopulation and the rest. While other reports have shown that a low CD4+ count is characteristic of low antibody response subpopulations [8], differences in the thresholds used to define these subpopulations cannot be overlooked. The third booster vaccination enhanced NAb titers against Wuhan and VOCs, in agreement with previous reports [31,32]. However, low NAb participants consistently exhibited significantly lower titers against all the VOCs tested, including two who showed no detectable change in VOC neutralization despite third booster vaccination. Although HIV uninfected subpopulations of low-NAb participants have shown similarly low titers post-booster [33], we could not establish whether participants showing no detectable change in NAb titers against VOCs post-booster were limited to PLWH or could also be found in the general population, given the lack of a healthy control group in this study. Nevertheless, our findings highlight the need for continuous monitoring of low-NAb subpopulations and their responses to booster vaccination in larger cohorts of PLWH.

Our investigation of host factors associated with NAb titers showed that age negatively correlated with NAb titers against Wuhan following both two-dose and third booster vaccination regimens. Our findings are consistent with previous reports from cohorts of PLWH from other countries [4,16] and HIV-uninfected healthcare workers from Japan [34,35]. Aging with HIV has been shown to accelerate immune senescence and could therefore limit the ability of older PLWH to mount robust antibody responses to de novo antigens [36,37] such as COVID-19 vaccination in this case. Given that the proportion of older PLWH is projected to increase significantly by the end of 2030 [38], our findings bring attention to the vulnerability of this subpopulation and call for their prioritization in future booster vaccination regimens.

On the other hand, we found that CD4^+^ count did not correlate with NAb titers against Wuhan after two-dose vaccination, a finding that is in line with previous reports [4,14,39] but also in contrast to some other reports [5,7]. It is, however, intriguing that our age subgroup analysis revealed that NAb titers in younger participants (≤48 years) positively correlated with the CD4^+^ count in participants with CD4^+^ counts within the subnormal range (≤500 cells/µL). Although we could not confidently ascribe the observed age-dependent association to differences in actual age or age-associated immune phenotypes, comorbidities, and coinfections, these findings highlight how the heterogeneity of clinical parameters and markers of immune reconstitution across cohorts could impede the building of consensus on the most vulnerable subpopulations of PLWH. As such, classifications of cohorts based on immune reconstitution markers, clinical and demographical parameters, may reveal a broader spectrum of host factor interactions with vaccine responses and help in building a consensus towards identifying the most at-risk subpopulations of PLWH for targeted vaccination strategies.

Our study was, however, not without limitations. First, the lack of an HIV-uninfected control group limits a direct comparison in NAb responses between PLWH and the general population. Also, our experimental approach relied on IgG neutralization thereby excluding other non-dominant isotypes, such as IgA and IgM, but with equally potent neutralization properties. Furthermore, considering that our cohort was 97.5% male, and differences in immune responses to vaccination may be present between males and females, further research is needed to understand the potential role of sex in NAb responses in PLWH. Additionally, the duration between vaccination and sample collection varied considerably across the cohort. Finally, our study was also limited by its small sample size. Nonetheless, our study contributes to a growing body of literature on factors associated with NAb responses to COVID-19 vaccination in PWLH. Our findings highlight an overall immune-boosting effect of monovalent third booster vaccination against Wuhan and VOCs. We identified differential host immune associations with NAb titers in different age subgroupings; however, further research in larger cohorts is needed to elucidate this observation.

## Figures and Tables

**Table 1:** Demographic and clinical characteristics of COVID-19-vaccinated PLWH.

2-Dose Vaccinated Participants (*n* = 82)	
Age in years, median (IQR)	48 (40–56)
Male, *n* (%)	80 (98)
mRNA vaccine received ^c^	
BNT162b2, *n* (%)	48 (71.6)
mRNA-1273, *n* (%)	19 (28.4)
Days since 2-dose vaccination	53 (27–83)
Receiving ART, *n* (%)	82 (100)
Pre-ART HIV viral load in log copies/mL, median (IQR)	4.8 (3.1–5.2) ^a^
Undetectable HIV viral load (at sample collection) *n*, (%)	74 (94%) ^b^
Pre-ART CD4^+^ count in cells/µL, median (IQR)	211 (28–376) ^a^
CD4^+^ count (at sample collection) in cells/µL, median (IQR)	470 (314–643) ^a^
CD4^+^/CD8^+^ ratio (at sample collection), median (IQR)	0.9 (0.4–1.1) ^a^
hsCRP (at sample collection) in mg/dL, median (IQR)	0.12 (0.05–0.3) ^c^
**3rd Booster Vaccinated Participants (*n* = 18) ^d^**	
Age in years, median (IQR)	49 (36–66)
Male, *n* (%)	18 (100)
mRNA booster received ^b^	
BNT162b2, *n* (%)	5 (33.3)
mRNA-1273, *n* (%)	10 (66.7)
Days since 3rd booster vaccination, median (IQR)	51 (37–70)
Days from 2-dose to 3rd booster vaccination, median (IQR)	207 (196–215)

ART, antiretroviral therapy; IQR, interquartile range; hsCRP, high-sensitivity C-Reactive protein. ^a^ Data missing for 13 participants; ^b^ data missing for 3 participants; ^c^ data missing for 15 participants; ^d^ 18/82 participants who received 3rd booster vaccination had their longitudinal samples obtained.

**Table 2:** Correlates of neutralizing antibody (NAb) titers (reciprocal IgG IC_50_) against SARS-CoV-2 following 2-dose vaccination expressed as Spearman’s r coefficients.

Variables	Bivariate	Multivariate ^a^
Age	−0.42 **(*p* = 0.001)**	−0.33 **(*p* = 0.007)**
Days since 2-dose vaccination	−0.45 **(*p* = 0.001)**	−0.34 **(*p* = 0.005)**
Pre-ART viral load (copies/mL)	0.21 (*p* = 0.087)	0.14 (*p* = 0.266)
Pre-ART CD4^+^ count (cells/µL)	0.18 (*p* = 0.141)	−0.05 (*p* = 0.691)
CD4^+^ count (at sample collection) (cells/µL)	0.28 **(*p* = 0.017)**	0.17 (*p* = 0.158)
CD4^+^/CD8^+^ ratio (at sample collection)	0.15 (*p* = 0.204)	0.04 (*p* = 0.767)
hsCRP (at sample collection) (mg/dL)	−0.16 (*p* = 0.193)	−0.18 (*p* = 0.158)

NAb, neutralizing antibody; hsCRP, high sensitivity C-reactive protein. ^a^ Multivariate analysis by nonparametric partial Spearman correlation analysis. *p* < 0.05 in boldface.

## Data Availability

All data generated and analyzed are included in this article. Additional data will be provided following a reasonable request to the corresponding author.

## Supplementary Materials

The following supporting information can be downloaded at https://www.mdpi.com/article/10.3390/v16040555/s1, Figure S1: Validation of polyclonal IgG neutralization assay.
