# Supplementary material for: Factors Associated with Neutralizing Antibody Responses following 2-Dose and 3rd Booster Monovalent COVID-19 Vaccination in Japanese People Living with HIV
Source: Viruses. 2024 Apr 2;16(4):555. doi: 10.3390/v16040555 (PMC11053946; doi:10.3390/v16040555)

Supplementary Figure S1

Pseudovirus neutralization assay was established by the use of 293T Cells transiently expressing ACE2/TMPRSS2. Pseudovirus infectivity was enhanced by the truncation of 19 amino acids from the C-terminal end of the parental Wuhan spike sequence to generate a variant designated as (CΔ19 spike) (panel A). Pseudovirus concentration was quantified as a function of p24 while infectivity is expressed as NunLuc luminescence.

Plasma from 3 representative PLWH on ART exhibited unspecific inhibition against the HIV lentiviral backbone pseudotyped by VSV-G envelope, MuLV envelope, and Wuhan spike (panel B). However, purification of polyclonal IgG showed Wuhan Spike-specific inhibition against which the participants were vaccinated.

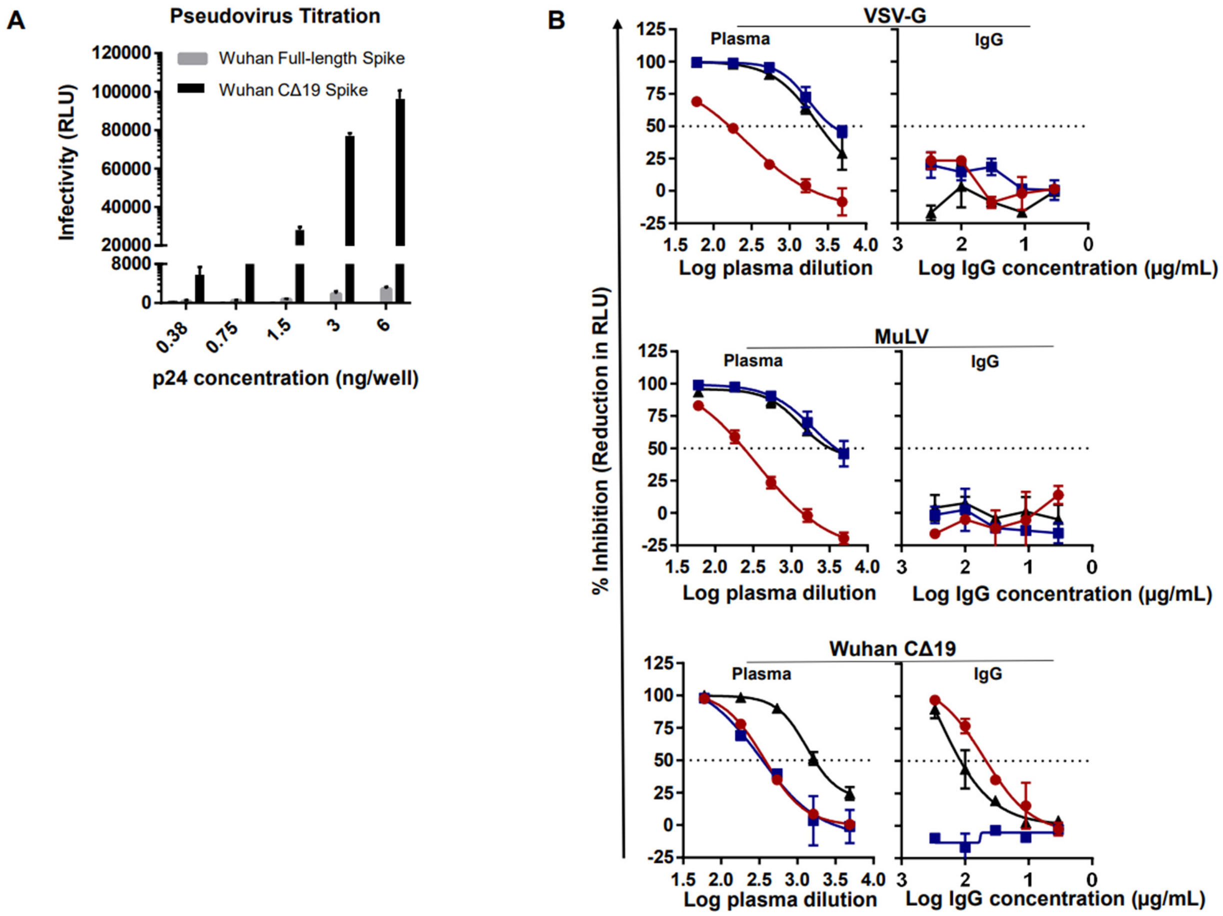

Supplement: Supplementary file 1 [file viruses-16-00555-s001.zip › viruses-2888438-supplementary.pdf]
